# A technical review of dynamic brain functional imaging combined with EEG spectral parameters in the screening of early-stage brain injury

**DOI:** 10.3389/fmedt.2026.1960562

**Published:** 2026-10-16

**Authors:** Hongmei Ruan, Liqing Liao, Yuan Zhang, Zicai Liu, Qiang Wu

**Affiliations:** 1 Department of Rehabilitation Medicine, Shaoguan University Affiliated Hospital (Shaoguan First People's Hospital), Shaoguan, Guangdong, China; 2 School of Rehabilitation Medicine, Gannan Medical University, Ganzhou, Jiangxi, China

**Keywords:** 1/f aperiodic component, dynamic functional magnetic resonance imaging, early brain injury, EEG spectral slowing, large-scale neural dynamics, multimodal screening

## Abstract

**Background:**

Early-stage brain injury encompasses subtypes such as mild traumatic brain injury (mTBI), silent stroke and subclinical brain injury. It is characterised by negative imaging findings, subtle symptoms and a high risk of long-term neurological deficits, presenting a key challenge in the early screening and prevention of neurological diseases. Traditional imaging techniques, such as structural Magnetic Resonance Imaging (MRI) and Computed Tomography (CT), can only identify organic morphological lesions. Their detection rate for early micro-injuries at the level of neuronal electrophysiology and brain network function is extremely low. Consequently, these techniques struggle to meet the clinical needs for early screening, assessment, and intervention.

**Methods:**

Electroencephalography (EEG) spectral changes (including conventional spectral slowing and 1/f aperiodic abnormalities) and large-scale dynamic neural network disruption across the entire brain are core pathological features of early brain functional damage; they may serve as indirect correlates of microscopic pathological changes such as an imbalance in neuronal excitation-inhibition and abnormalities in the efficiency of brain information transmission. Based on this, this paper proposes a Dynamic-Spectral Coupling (DSC) framework, which proposes spectral abnormalities as a potential upstream mechanism associated with network reorganisation. Within this framework, this narrative review summarizes dynamic functional magnetic resonance imaging (dynamic fMRI), EEG spectral analysis and multimodal fusion technologies, with a particular focus on elucidating the coupling mechanisms between EEG periodic and 1/f aperiodic spectral parameters and dynamic brain network indicators.

**Results:**

The review outlines the screening value and application advantages of multimodal fusion technologies in mTBI, occult cerebrovascular injury and subclinical brain injury. Furthermore, the paper summarises the current limitations of these technologies in terms of clinical translation, methodology and database development.

**Conclusions:**

Taking into account the clinical needs of neurorehabilitation, it proposes future development directions, including the development of lightweight devices, AI-assisted diagnosis, the establishment of standardised analytical systems and the translation of precision rehabilitation. The aim is to provide technical guidance and theoretical support for screening, risk stratification and rehabilitation planning in early-stage brain injury, while acknowledging that clinical utility remains to be established.

## 1 Introduction

### 1.1 Definition of early brain injury and challenges in clinical screening

In this review, “early brain injury” is used as a methodological umbrella concept rather than a biological construct. It denotes conditions in which functional or microstructural abnormalities may be present despite negative findings on conventional structural imaging. The included conditions—mTBI, silent stroke, and subclinical brain injury—differ substantially in pathophysiology, temporal evolution, and clinical trajectory (1). A shared analytical framework does not imply that they constitute a single disease entity (2).

Globally, more than 50 million people suffer from traumatic brain injury each year (3). The global burden of neurological disorders has risen substantially in recent decades. According to the Global Burden of Disease Study 2023, neurological conditions are among the leading causes of death and disability worldwide, with 292 causes of death analysed across 204 countries and territories and 660 subnational locations (4). This growing burden underscores the urgent need for scalable screening approaches for early-stage brain injury. Such injuries are prevalent among sports injury patients, individuals at high risk of cerebrovascular disease, the elderly, and patients with mild cranial trauma. A common feature of these conditions is that functional pathological changes can occur in the early stages of the disease. These changes include neuronal dysfunction, abnormal brain network connectivity and microscopic white matter damage. However, conventional structural imaging examinations often fail to detect such abnormalities (5).

Currently, routine clinical Computed Tomography (CT) and structural Magnetic Resonance Imaging (MRI) are primarily used to detect morphological lesions such as haemorrhage, oedema and infarction; they have limited sensitivity to early pathological changes such as diffuse axonal injury, abnormalities in brain network function and microscopic white matter damage (6). A large number of patients continue to exhibit persistent neurological dysfunction, yet conventional structural imaging studies may remain negative. In addition to routine imaging, commonly used clinical methods for assessing brain injury include electroencephalography (EEG), diffusion-weighted imaging (DWI), functional magnetic resonance imaging (fMRI), serum biomarkers, and bedside assessments of pupil response and consciousness. These methods differ in terms of sensitivity, specificity, bedside accessibility and operational requirements, as shown in Figure 1. Among these, EEG offers the advantages of real-time bedside monitoring, non-invasiveness and the ability to perform dynamic follow-up examinations; however, it has limited spatial resolution and is susceptible to interference (7). fMRI provides accurate spatial localisation and can reflect changes in brain network function, but the examination is time-consuming and critically ill patients may find it difficult to cooperate (8); DWI is highly sensitive to early ischaemic changes but may still fail to detect minor lesions (9); serum biomarkers are convenient to sample and allow for dynamic follow-up, but their specificity is limited and they must be combined with imaging (10); bedside assessments of consciousness and pupil response are rapid and convenient, but are highly subjective and show no obvious early changes (11). The aforementioned individual methods are unable to comprehensively cover the multi-level pathological changes associated with early cerebral functional impairment; there is an urgent need to develop a multimodal combined assessment system. Although advanced imaging techniques such as diffusion tensor imaging (DTI) and fMRI can detect abnormalities in brain network connectivity and microstructure, providing new objective evidence for the identification of early cerebral functional impairment (12), their use as standalone methods remains limited.

**Figure 1:**
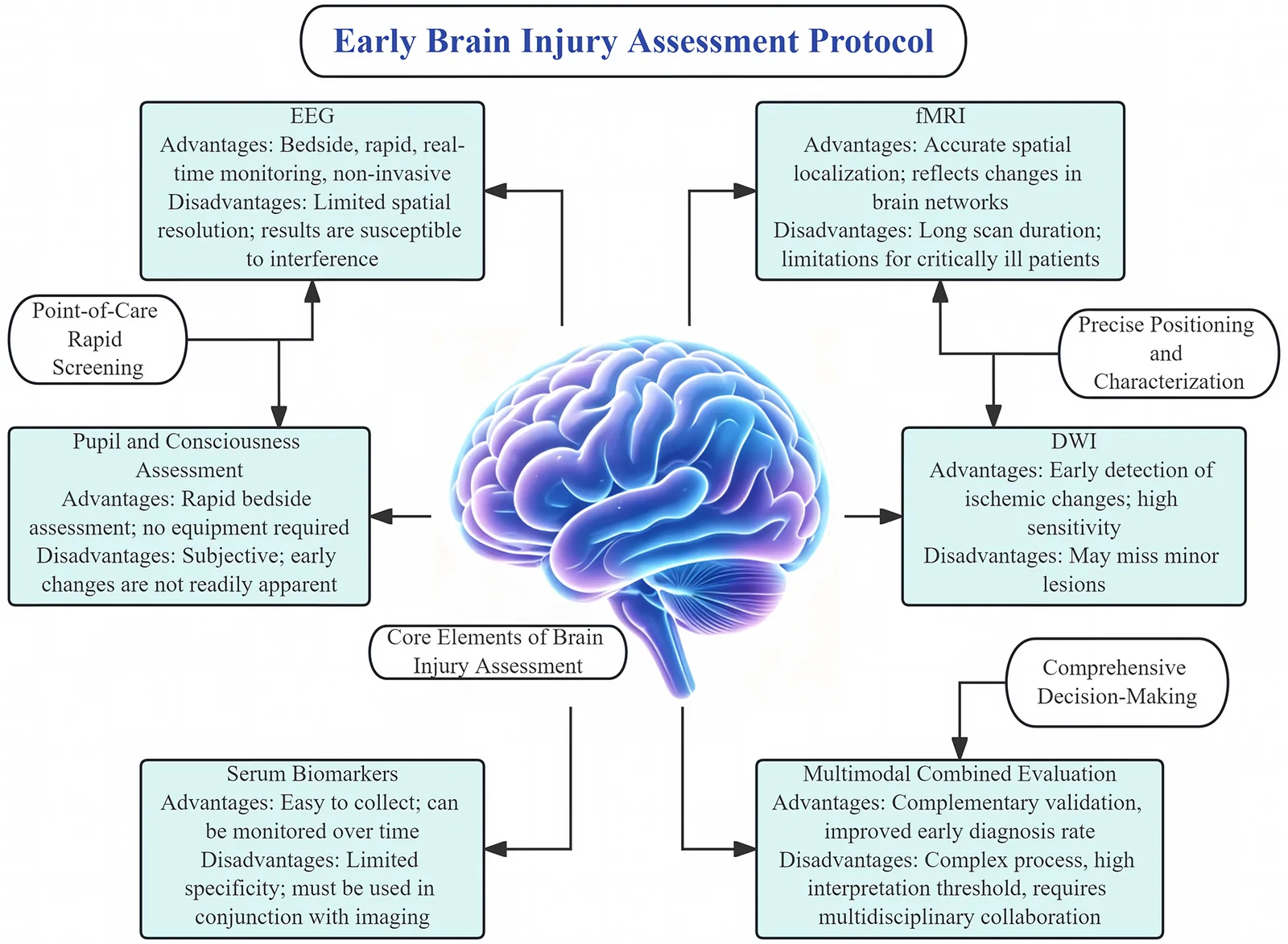
Commonly used tests and assessment methods for early-stage brain injury.

Apart from the subtle nature of the imaging findings, clinical symptoms in patients with early-stage brain injury are typically non-specific, often manifesting as mild dizziness, headaches, reduced concentration, cognitive fatigue, sleep disturbances and mood changes. These symptoms are easily overlooked by both patients and clinicians, resulting in a low rate of early detection (13, 14). A growing body of research indicates that, if not identified in a timely manner and provided with standardised intervention, some patients may experience persistent cognitive impairment, ongoing post-concussion symptoms, and neuropsychiatric impairments such as anxiety and depression (15). For patients with cerebral small-vessel disease and silent cerebral ischaemia, this may also increase the risk of long-term adverse outcomes such as cognitive decline, dementia and stroke, placing a sustained burden on public health and rehabilitation services (16). At present, there is a lack of a unified, objective screening and assessment system for early-stage brain functional impairment. Clinical diagnosis still relies primarily on symptom assessment, neuropsychological scales and routine structural imaging examinations, which struggle to objectively reflect changes in brain network function and the extent of damage (17). Therefore, the development of novel early-stage screening technologies based on objective biomarkers and multimodal brain function assessment is of significant clinical importance for improving diagnostic classification, evaluating treatment efficacy, and guiding rehabilitation interventions (18).

### 1.2 Key concepts: EEG spectral slowing, large-scale dynamic brain networks

EEG spectral slowing is one of the most common and relatively stable electrophysiological features of early brain injury (19). To understand this concept, it is first necessary to clarify the basic meaning of the EEG spectrum: the distribution curve of power as a function of frequency obtained by applying a Fourier transform to convert the raw EEG signal from the time domain to the frequency domain (20). Spectral slowing is primarily characterised by a reduction in the power of high-frequency oscillations (β and γ bands) in the EEG signal, whilst low-frequency oscillations (δ and θ bands) are relatively enhanced; this reflects dysrhythmia in neuronal discharge, reduced synaptic transmission efficiency, and an imbalance in cortical excitation–inhibition (21, 22). In recent years, with the development of parametric analysis of EEG power spectra, the 1/f aperiodic component within EEG power spectra has gradually become an important indicator in brain function research (23). Compared with traditional frequency-band power analysis, the 1/f parameter can effectively separate periodic oscillations from aperiodic background activity, more accurately reflecting the overall excitability of neuronal populations, synaptic background activity and the state of cortical information processing. It provides supplementary information, independent of traditional spectral indicators, for identifying early-stage abnormalities in brain function and is regarded as a promising new electrophysiological biomarker in research into brain injury and neurodegenerative diseases (24, 25).

However, brain injury manifests not only as local abnormalities in neuronal electrical activity but also involves the reconstruction of co-operative functions across multiple brain regions (26). Consequently, relying solely on EEG spectral measures makes it difficult to comprehensively reflect the network characteristics of brain functional impairment. Large-scale dynamic functional brain network analysis based on resting-state fMRI can characterise the dynamic changes in functional connectivity between brain regions over time at a whole-brain scale. Compared with traditional static functional connectivity analysis, it is better able to reveal dynamic characteristics such as brain network stability, state transition capacity and global information integration efficiency (27). A growing body of research indicates that early brain injury is not confined to localised brain regions, but rather constitutes a widespread functional dysregulation involving multiple large-scale brain networks, such as the default mode network (DMN), salience network (SN) and frontoparietal network (FPN). This is primarily manifested as weakened dynamic functional connectivity, abnormal network state transitions, impaired modular organisation and reduced global information transmission efficiency (28).

EEG spectral features and dynamic brain network metrics reflect brain function at different levels. EEG spectral features describe changes in the electrical activity and excitability of neuronal populations, constituting a microscopic temporal-scale electrophysiological metric. In contrast, dynamic brain network metrics reveal the reorganisation of functional connectivity between brain regions and the dynamic organisation of networks, constituting a macroscopic spatial-scale brain network metric (29). The two exhibit a high degree of pathological coupling and complementarity; multimodal joint analysis holds promise for enabling cross-scale assessment of early brain injury—from abnormal neuronal activity to the functional reorganisation of brain networks—thereby providing a new theoretical foundation and technical pathway for establishing a more objective and sensitive brain function screening system (30).

### 1.3 Purpose of the review

This paper focuses on the key technical bottlenecks facing functional screening for early-stage brain injury. Based on the theoretical framework of EEG spectral features (including 1/f aperiodic components) and the pathological mechanisms underlying dynamic brain network disruption, this narrative review summarizes the principles, biomarkers and applicable scenarios of single EEG spectral analysis and dynamic fMRI techniques. It elaborates in detail on the mainstream strategies for synchronous and asynchronous fMRI-EEG multimodal fusion and their diagnostic advantages, and analyses their clinical application value across three scenarios: mTBI, silent stroke and subclinical brain injury. Finally, it discusses key challenges such as clinical translation, algorithm standardisation and database construction. Based on this, this paper proposes, for the first time, the “Dynamic-Spectral Coupling (DSC)” analytical framework. Its core hypothesis is that local E/I imbalance, as reflected by EEG periodic components and 1/f aperiodic parameters, may serve as an upstream mechanism contributing to abnormal switching of whole-brain dynamic network states. The entire paper is structured around this framework, aiming to provide a theoretical basis for improving screening technologies for early-stage brain injury and facilitating their clinical translation into rehabilitation.

## 2 Core neural mechanisms of early brain injury

### 2.1 Imbalance in cortical excitatory-inhibitory activity as the core pathological basis

The cortical excitatory–inhibitory (E/I) balance is the cornerstone for maintaining normal brain electrical activity, information transmission and network coordination, and is primarily achieved through the dynamic regulation of glutamatergic excitatory neurons and γ-aminobutyric acid (GABA) inhibitory neurons (31). A key pathophysiological feature of early brain injury may be a diffuse disruption of the E/I balance that is not typically detectable by conventional structural imaging (32), although this remains a hypothesis and does not exclude concurrent structural or microstructural changes. Various early injury triggers (external impact, cerebral microcirculatory disorders, subclinical ischaemia-hypoxia) can directly lead to impaired function of inhibitory neurons and weakened synaptic inhibition, resulting in excessive excitation of cortical neurons and disrupted discharge rhythms (31). This imbalance in the E/I balance further induces a cascade of pathological reactions: abnormal neuronal discharges lead to disrupted energy metabolism and impaired mitochondrial function within the brain, which in turn reduces synaptic transmission efficiency and hinders information exchange between brain regions; simultaneously, this disrupts the modular, coordinated regulatory capacity of core cognitive networks (33)—such as the DMN, the SN and the FPN—manifesting as dynamic connectivity abnormalities, including increased network switching rates and shortened state dwell times. These pathological changes may occur prior to structural damage. They may serve as the initiating factor for early brain functional impairment. Furthermore, they constitute the fundamental pathological basis for EEG spectral abnormalities and dynamic brain network disruptions, providing a core pathological basis for functional imaging and EEG screening techniques (34).

Figure 2 provides a schematic overview of the pathophysiological mechanism underlying the aforementioned “dynamic-spectral coupling”. As shown in the figure, various early triggers of brain injury (external impact, microcirculatory disorders, subclinical shock) first disrupt the excitatory-inhibitory balance between glutamate and GABA (35), leading to increased cortical excitability and reduced inhibitory activity. At the EEG level, this microscopic electrophysiological disruption manifests as spectral slowing and a flattening of the 1/f slope (36); at the brain network level, it manifests as a decline in the dynamic connectivity stability of the three core networks—the DMN, SN and FPN—characterised by increased switching rates and shorter dwell times (35). This hypothesised pathway illustrates the cascading pathological logic from “microscopic E/I imbalance” to “macroscopic network reorganisation”, providing a visual framework for mechanistic explanations of subsequent multimodal fusion screening technologies.

**Figure 2:**
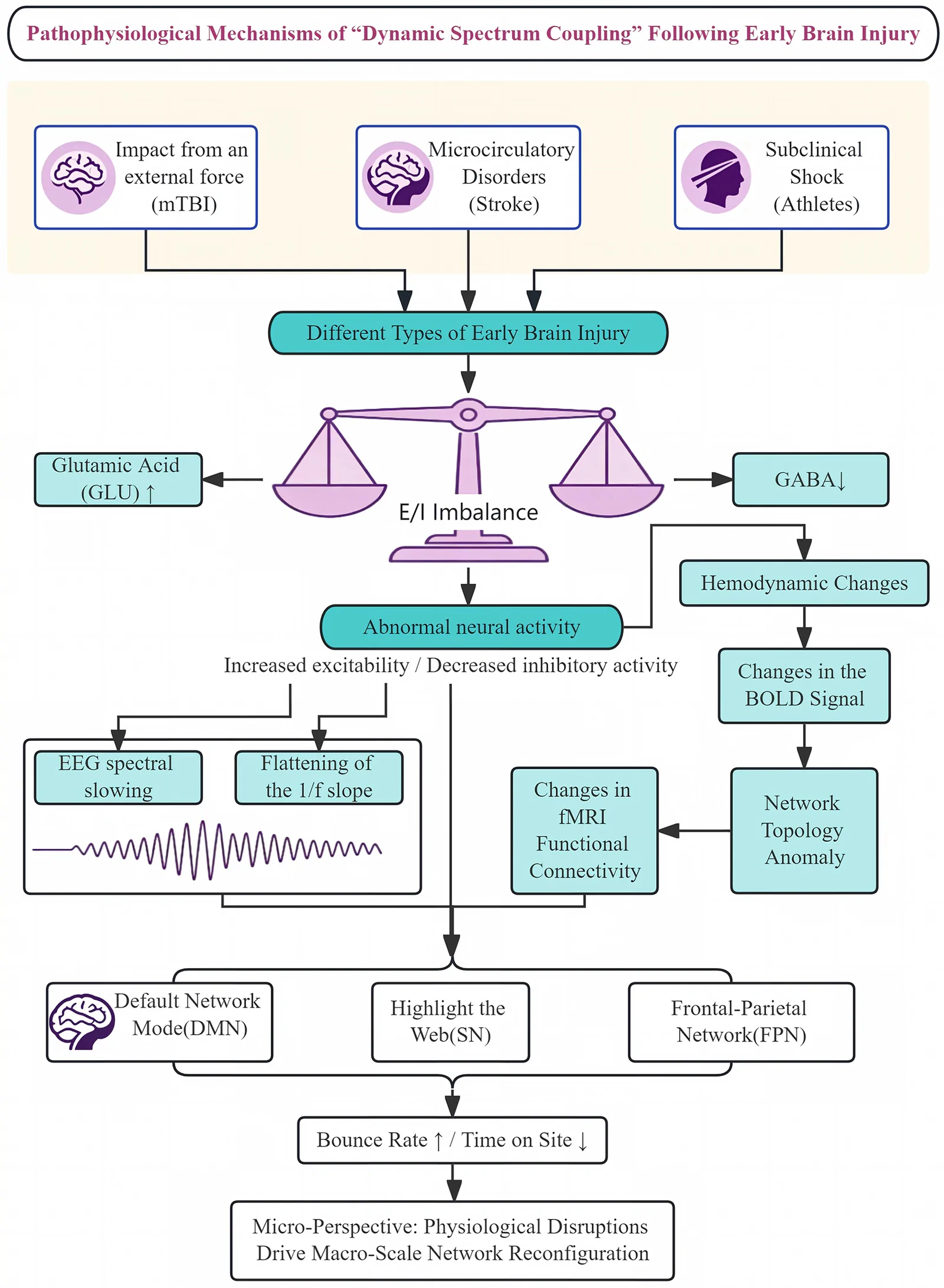
Pathophysiological mechanisms underlying dynamic-spectral coupling in early brain injury.

### 2.2 Mechanisms underlying EEG spectral abnormalities: periodic slowing and aperiodic attenuation

Before discussing these mechanisms, it is important to distinguish two related but conceptually distinct terms. We use “spectral slowing” to refer to conventional band-power changes (increased low-frequency and decreased high-frequency power), whereas “aperiodic flattening” refers to a reduction in the 1/f exponent. These are conceptually distinct and should not be conflated.

EEG signals consist of periodic oscillations and a 1/f aperiodic background component; these reflect, respectively, the rhythmic firing activity of neurons and the overall excitatory state of neural populations, and together determine the fundamental characteristics of the EEG power spectrum (37). Recent studies have shown that early brain injury not only causes classic periodic spectral abnormalities but is also accompanied by changes in the 1/f aperiodic component; these two types of spectral features collectively may reflect neuronal dysfunction and an imbalance in the homeostasis of brain networks, providing potential electrophysiological evidence for early brain functional impairment (38).

Abnormalities in the periodic spectrum are primarily manifested as an increase in the power of low-frequency oscillations (δ and θ bands) and a decrease in the power of high-frequency oscillations (β and γ bands), i.e., typical EEG spectral slowing (22). The underlying mechanism is mainly related to abnormalities in neuronal discharge synchrony caused by an imbalance in the excitation-inhibition (E/I) balance. A decline in the function of inhibitory neurons leads to abnormal synchronous discharge of cortical neurons, resulting in a significant enhancement of low-frequency slow-wave activity; at the same time, due to impaired energy metabolism, reduced synaptic transmission efficiency and compromised neuronal information encoding capacity, high-frequency oscillatory activity gradually weakens, ultimately manifesting as impaired attention, executive function and cognitive processing abilities (39). Research has shown that a slowing of the electroencephalographic spectrum is a sensitive indicator of early functional impairment in brain tissue and is closely correlated with the severity of brain injury and the recovery of neurological function; it has therefore become one of the most commonly used electrophysiological indicators in the assessment of brain dysfunction (40).

In recent years, the 1/f aperiodic component has gradually emerged as an important area of research in EEG spectral analysis. Unlike periodic oscillations, the 1/f aperiodic background signal reflects the overall discharge characteristics of neuronal populations; its exponent and offset may serve as indirect correlates of cortical excitability, synaptic input and the E/I balance, although the relationship is not specific (41). Therefore, 1/f parameters should be interpreted as indirect correlates of cortical dynamics rather than direct quantitative proxies for E/I balance. E/I imbalance remains a biological hypothesis, whereas 1/f slope is an EEG-derived summary measure influenced by multiple physiological and non-physiological factors. The power spectrum of a normal EEG typically exhibits stable power-law decay characteristics, whereas diffuse neuronal dysfunction caused by early-stage brain injury can lead to a reduction in the 1/f aperiodic index (flattening of the slope) and abnormal offset values, suggesting a decline in the organisation of cortical neural activity and impaired neural network homeostasis (42). Compared with traditional frequency-band power analysis, aperiodic parameters can effectively reduce the confounding effects associated with fixed frequency-band divisions and enhance the ability to quantitatively characterise changes in brain functional states; they are therefore regarded as a novel EEG biomarker with significant application potential (43). Abnormalities in periodic oscillations, together with alterations in the 1/f aperiodic component, constitute key features of microscopic electrophysiological abnormalities in early-stage brain injury, providing a new theoretical foundation for ultra-early screening of brain injury and multimodal neuroimaging fusion analysis (42). These two phenomena should not be conflated, as they reflect distinct underlying mechanisms.

It is worth noting that changes in the slope of the 1/f aperiodic component not only reflect the E/I balance (44) but are also closely related to the critical dynamics of the nervous system (45). In recent years, researchers have sought to understand aperiodic EEG activity within the broader framework of scale-free neuronal dynamics and brain criticality (46). EEG measures related to criticality provide an alternative approach to describing large-scale neuronal dynamics that goes beyond traditional frequency-band measurements (46), and longitudinal studies have confirmed that changes in critical brain dynamics are associated with cognitive function (47). This perspective complements the discussion in this paper regarding 1/f activity and network steady-state behavior. For example, Tseriotis et al. (48). proposed that criticality in EEG could serve as a monitoring biomarker for cognitive impairment. Introducing this framework helps link micro-level aperiodic activity to macro-level network reorganization in early brain injury and provides a theoretical bridge to the discussion of dynamic brain network disruptions in the next section.

### 2.3 Characteristics of large-scale dynamic brain network disruption and their coupling with spectral parameters

The brain is a complex dynamic system composed of multiple interacting large-scale functional networks, whose functional connectivity patterns are characterised by persistent fluctuations and dynamic reorganisation. Under normal conditions, brain networks are capable of maintaining relatively stable dynamic connectivity patterns, rational modular organisation, and efficient information integration (49). Disruptions in the E/I balance and local electrophysiological abnormalities can further affect the synchrony of neural activity across brain regions, gradually manifesting as functional reorganisation of large-scale brain networks (50). Key manifestations include reduced stability of dynamic functional connectivity, abnormal network state transitions, decreased modularity of brain networks, reduced global efficiency, as well as local hyperconnectivity and weakened long-range connectivity (51). Dynamic functional connectivity analysis can describe the connectivity patterns of brain networks over time and the processes of network state transitions; compared with static analysis, it is better suited to identifying transient and intermittent functional abnormalities (52).

Evidence suggests a close coupling between EEG spectral parameters and dynamic brain network indicators; multiple studies have found that both can reflect brain functional abnormalities at different scales and exhibit a certain degree of consistency (53, 54). At the micro level, EEG spectral slowing and abnormalities in the 1/f aperiodic slope reflect abnormalities in the firing rhythms and excitability of neurons within individual brain regions; at the macro level, dynamic brain network disruption may represent the overall manifestation of a loss of coordination in neuronal electrical activity across multiple brain regions (42). Numerous studies have found that enhanced low-frequency EEG activity and abnormalities in 1/f aperiodic parameters are frequently accompanied by alterations in the dynamic connectivity patterns of the DMN, SN and FPN; however, results obtained from different diseases and using different research methods still exhibit some variation, whilst the global information transmission efficiency of the network shows a significant positive correlation with spectral parameters (6). Consequently, the combined analysis of EEG spectral features and dynamic brain network parameters holds promise for establishing a multi-scale assessment framework linking “microscopic neurophysiology” with “macroscopic brain network function”, thereby providing a crucial theoretical foundation for the application of EEG-fMRI multimodal fusion technology in the screening of early-stage brain injury.

## 3 Single-modality techniques and their screening value

### 3.1 EEG spectral analysis: conventional frequency band parameters, 1/f aperiodicity index, and microstate dynamics

EEG spectral analysis is a core technique for the electrophysiological screening of early brain injury. It offers the advantages of being non-invasive, low-cost, high-temporal resolution and capable of dynamic monitoring. It primarily comprises three core assessment indicators: conventional frequency band parameters, the 1/f aperiodicity index, and EEG microstate dynamics (55). Conventional frequency band parameters focus on the absolute power, relative power and frequency band ratios (θ/α, δ/α) of the δ, θ, α, β and γ bands. Among these, an elevated θ/α ratio is the most classic spectral indicator of early brain injury; it effectively reflects the degree of impairment in cognitive function and is widely used in scenarios such as the initial screening for mTBI and the assessment of brain fatigue (56, 57). However, conventional frequency-band parameters suffer from a lack of specificity and are susceptible to interference from confounding factors such as age, sleep state, mood and medication; when used in isolation, they struggle to distinguish between mild pathological injury and physiological fluctuations in brain function.

The 1/f aperiodic index is a breakthrough biomarker that has emerged in recent years in the field of neuroelectrophysiology. Its commonly used computational framework is FOOOF (Fitting Oscillations & One Over F), which employs parametric modelling to decompose the EEG power spectrum into periodic oscillations and aperiodic background components (23). Key parameters include spectral slope and spectral offset, which can independently isolate interference from periodic oscillatory signals and directly quantify the excitability of neuronal populations and the level of synaptic activity. In early-stage brain injury, the 1/f aperiodic index mitigates the influence of periodic oscillations and individual variability inherent in traditional frequency-band analysis, whilst capturing background neurodynamic abnormalities associated with mTBI (40, 58). It thus provides a new candidate indicator for the early detection of functional impairment in patients with normal structural imaging, thereby addressing the limitations of conventional frequency-band parameters (59). EEG microstate dynamics, meanwhile, focuses on dynamic changes in the spatiotemporal distribution of EEG activity. By quantifying microstate duration, occurrence frequency and transition probabilities, it characterises the spatiotemporal stability of whole-brain EEG activity. Patients with early-stage brain injury often exhibit disrupted microstate transitions and abnormal proportions of dominant microstates, reflecting impaired temporal regulatory function of the brain network (28).

Overall, EEG spectral analysis techniques can accurately capture microscopic electrophysiological abnormalities in early-stage brain injury, with a temporal resolution of up to the millisecond level, making them suitable for rapid bedside screening and dynamic follow-up; however, their spatial resolution is insufficient to precisely localise the brain regions and network abnormalities corresponding to the injury (60).

### 3.2 Dynamic brain functional imaging techniques: key indicators of dynamic functional connectivity and network stability

Dynamic fMRI technology utilises the temporal variations in blood oxygen level-dependent (BOLD) signals to construct a whole-brain dynamic functional connectivity network. This overcomes the limitations of traditional static fMRI, which can only reflect average brain functional states (49), and serves as a core imaging technique for characterising macroscopic network disruptions in early-stage brain injury. Its core screening indicators include dynamic functional connectivity strength, connectivity stability, network modularity, global/local efficiency, and spatio-temporal coefficient of variation (61). Early-stage brain injury does not present with obvious signal abnormalities in specific local brain regions; rather, it manifests primarily as abnormalities in the dynamic regulatory function of the whole-brain network. Specifically, this involves a decline in the dynamic connectivity stability of core cognitive networks (62, 63)—such as the DMN, the frontoparietal network and the SN—blurred modular differentiation within brain networks, reduced efficiency of collaborative transmission between distant brain regions, and the emergence of abnormal hyperconnectivity in local brain regions (6).

Dynamic fMRI possesses ultra-high spatial resolution, enabling the accurate localisation of core brain regions and pathways involved in network disruption (64), the quantification of the extent of damage across the entire brain and within subnetworks, and the provision of visualised evidence of macroscopic functional impairment in early-stage brain injury. Numerous studies have confirmed that patients with mTBI or subclinical cerebral small-vessel disease who test negative on structural MRI already exhibit significant abnormalities in dynamic fMRI metrics (64); furthermore, the degree of network disruption is significantly correlated with patients' cognitive function and level of recovery, making it suitable for injury stratification and prognostic assessment. However, dynamic fMRI has low temporal resolution and is unable to capture rapid changes in neuronal electrical activity at the millisecond level (65). Furthermore, due to long scan times, expensive equipment and complex testing procedures, it is difficult to implement rapid bedside screening and dynamic monitoring (66).

### 3.3 Comparison of the advantages and disadvantages of monomodal techniques and their applicable scenarios

EEG spectral analysis and dynamic fMRI, as two core monomodal screening techniques, are clearly complementary in terms of temporal resolution and spatial localisation capabilities. A summary of the comparison between EEG spectral analysis and dynamic fMRI in terms of temporal/spatial resolution, portability, cost, and screening advantages is presented in Table 1.

**Table 1:** A comparison of core performance characteristics between EEG spectral analysis and dynamic fMRI monomodal techniques.

Dimension	EEG spectrum analysis (qEEG)	Dynamic fMRI (Dynamic functional connectivity, DFC)
The Essence of Signal Detection	Direct recording of synchronised electrical activity in neuronal clusters to quantify spectral power in the δ/θ/α/β frequency bands and 1/f aperiodic components of micro-electrophysiological abnormalities	Indirect detection of BOLD haemodynamic signals, reflecting large-scale network fluctuations in blood oxygenation across the entire brain
Temporal resolution	At the millisecond level, capable of capturing transient neuronal discharge oscillations	Second-level (limited by the blood flow response function); unable to detect transient electrophysiological disturbances
Spatial resolution	Poor accuracy; constraints arising from scalp volume conduction and inverse problems make it difficult to precisely localise deep or localised areas of brain damage	High (1–3 mm), enabling precise localisation of lesion circuits—such as the default network, the frontoparietal network and the SN—as well as subcortical nuclei
Portability	Lightweight, non-invasive, continuous bedside monitoring; simple to operate and quick to perform	Large MRI scanners, with their enclosed scanning chambers, cannot be operated at the patient's bedside; their use is restricted by agitated or critically ill bedridden patients.
Testing Costs and Repeatability	Cost-effective; supports repeat testing during the acute phase, long-term follow-up during rehabilitation, and large-scale population screening	It is expensive, each test takes a long time, and it is difficult to carry out repeated follow-up screening tests
Advantages of TBI Assessment	Detects microscopic electrophysiological disturbances in neurons, making it suitable for rapid initial screening in the early stages of mTBI, follow-up assessments of mild conditions after exercise, and dynamic monitoring of rehabilitation outcomes.	Visualises imbalances in the reconstruction of macro-level whole-brain networks; suitable for the diagnosis support of CT/MRI-negative occult brain injuries, research into injury mechanisms, and the prediction of long-term prognosis
Key limitations	Unable to reveal macroscopic functional dissociation in whole-brain networks following brain injury; assessment is limited to a single dimension	It can only reflect indirect changes in blood flow; it lacks information on the intrinsic electrical activity of neurons, and there is a discrepancy between the scanning environment and actual physiological brain activity.
Common defects in single-modality imaging	As these approaches can only cover a single pathological level—either “microscopic electrophysiology” or “macroscopic networks”—and are unable to fully characterise the cross-scale pathological cascades of early brain injury, this has led to the development of fMRI-EEG multimodal fusion technology.	

## 4 Dynamic fMRI-EEG spectral multimodal fusion technology

We present the DSC framework as a testable mechanistic hypothesis rather than an established causal model. Simultaneous EEG-fMRI improves temporal correspondence, but temporal simultaneity alone does not establish causality. Multimodal fusion technology is a promising approach to overcoming the limitations of single-modality assessment and improving early screening of brain injury. EEG spectral analysis can capture abnormalities in neuronal electrical activity at the millisecond level, but lacks spatial localisation capability; dynamic fMRI can precisely characterise the spatial patterns of whole-brain network reorganisation, but is limited in temporal resolution and difficult to perform at the bedside. The two modalities are naturally complementary in the spatio-temporal dimensions (67); however, fusion is not merely a simple superposition, but rather an attempt to test a hypothesised mechanistic link between “microscopic rhythmic disturbances” and “macroscopic network drifts”. As discussed in Section 1.3 above, the Dynamic-Spectral Coupling (DSC) framework proposed in this paper is centred precisely on this hypothesised relationship. Building upon this framework, this section summarizes the technical system and potential screening applications of dynamic fMRI-EEG multimodal fusion across three levels: acquisition paradigms, fusion strategies, and joint biomarkers.

Based on the aforementioned DSC framework, multimodal fusion strategies can be categorised into three levels according to the depth of data integration, as shown in Figure 3. Feature-level fusion involves the separate extraction of EEG spectral features and dynamic fMRI network features; these are then combined through feature concatenation and Canonical Correlation Analysis (CCA) to construct joint biomarkers. This approach offers the highest level of technical maturity and the greatest clinical accessibility (68); source imaging fusion utilises source imaging algorithms such as sLORETA and Beamformer to invert scalp EEG signals to cortical source regions, which are then spatially registered against fMRI spatial activation maps to achieve precise correspondence between cortical electrical activity and functional brain networks; this approach is suitable for localising complex cases with negative structural imaging findings (69); Deep learning fusion uses raw or preprocessed EEG time-frequency spectra and fMRI dynamic connectivity matrices as direct inputs (70). Through models such as CNNs, GNNs and Transformers, it automatically uncovers deep cross-modal correlations, outputting lesion grading or risk probabilities; this represents the future direction for achieving fully automated intelligent screening.

**Figure 3:**
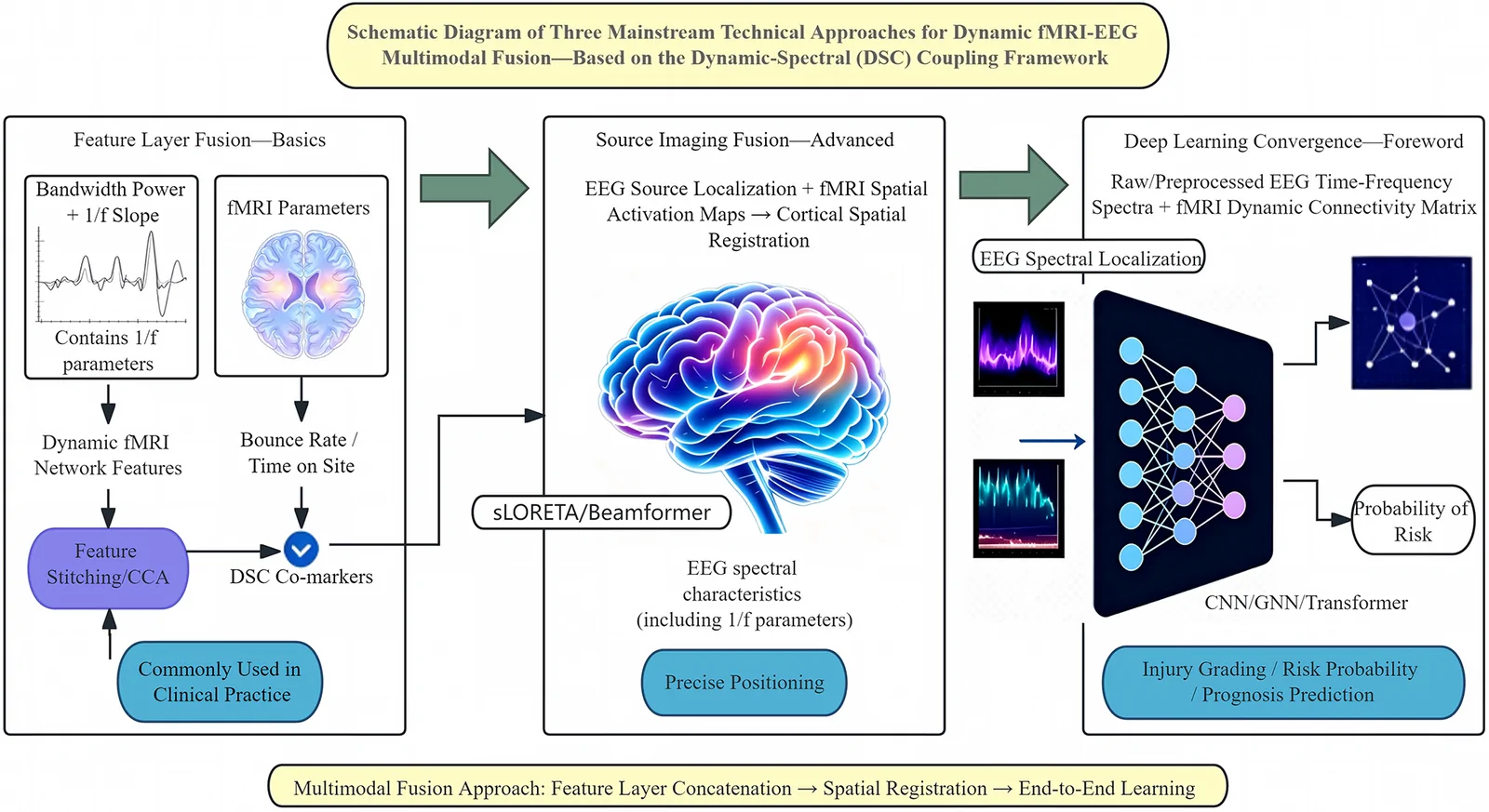
Roadmaps for three technical approaches to multimodal fusion.

### 4.1 Synchronous/asynchronous multimodal acquisition paradigms

Dynamic fMRI-EEG multimodal fusion technology is categorised into two core paradigms—synchronous and asynchronous acquisition—based on the timing of data collection, catering to different clinical screening and research scenarios.

The synchronous acquisition paradigm involves the simultaneous collection of EEG signals during an fMRI scan. This enables real-time matching and precise spatiotemporal alignment of cerebral oxygen metabolism signals with neuronal electrical activity signals (71). This paradigm minimises temporal discrepancies and may facilitate the construction of a real-time association model linking “electrical activity–oxygen metabolism–network connectivity”. It is capable of capturing transient, synchronous electrophysiological and network abnormalities in early-stage brain injury, and serves as the “gold standard” technical approach for validating causal chains within the DSC framework. However, synchronous acquisition faces three technical barriers: firstly, the gradient artefacts induced on EEG electrodes by MRI gradient field switching can reach amplitudes of tens of millivolts, completely drowning out microvolt-level EEG signals (72); secondly, ballistocardiogram (BCG) artefacts caused by cardiac pulsations overlap with the δ/θ frequency bands, severely compromising the accuracy of the low-frequency spectrum (73); thirdly, MRI scan noise (approximately 90–110 dB) may alter the patient's state of alertness, affecting resting-state *α* rhythms and BOLD fluctuations in the DMN (74). These technical hurdles mean that the quality of EEG data obtained via synchronous acquisition remains inferior to that from dedicated EEG equipment; furthermore, the high cost of specialised MRI-compatible electrodes and anti-interference systems currently limits their use primarily to centres conducting mechanistic research (75).

The asynchronous acquisition paradigm involves conducting EEG recordings and dynamic fMRI scans at different time points, whilst strictly controlling the testing environment, patient condition and baseline conditions to ensure consistency, and achieving the matching and fusion of the two types of data through post-processing (76). This paradigm is simple to operate, offers strong equipment compatibility and is free from MRI environmental interference (77); it effectively mitigates disruptive factors such as electromagnetic noise and motion artefacts associated with synchronous acquisition, making it suitable for large-scale clinical screening and routine testing. However, the assumption that physiological states are comparable across separate sessions requires empirical validation. State dependence (e.g., arousal, alertness, circadian rhythms, medication effects) and temporal instability may reduce cross-modal correspondence (78). Alternative strategies include state-matched sampling, repeated measures, hybrid designs with simultaneous EEG-fMRI in a subset, and physiological monitoring to align states (79). Future studies should quantify how temporal instability affects multimodal fusion. Although minor temporal discrepancies may occur, standardised data pre-processing, baseline correction and state matching may help meet the accuracy requirements for clinical screening, injury stratification and prognostic assessment; it is therefore a pragmatic first step for clinical translation, provided that state dependence is carefully addressed and, where possible, validated against simultaneous recordings.

The synchronous paradigm is suitable for validation studies of the DSC mechanism and for analyses of cognitive states requiring precise temporal alignment; however, due to equipment costs and operational complexity, it is difficult to promote as a routine screening tool (80). Although the asynchronous paradigm sacrifices millisecond-level temporal synchronisation, it balances clinical feasibility, cost-effectiveness and screening accuracy, making it a feasible acquisition scheme for the clinical translation of multimodal screening for early brain injury, provided that state comparability is ensured. For critically ill patients in the A&E department or ICU who are unable to undergo fMRI scans, bedside EEG monitoring may be used as a standalone method, with dynamic fMRI assessment carried out once the patient's condition has stabilised (81), thereby constituting a stepwise, asynchronous fusion strategy of “EEG initial screening → dynamic fMRI precise localisation”.

### 4.2 Mainstream fusion strategies: feature-level fusion, source imaging fusion and deep learning fusion

Current dynamic fMRI-EEG multimodal fusion can be categorised into three levels, progressing gradually from traditional data-driven approaches to intelligent causal inference.

The first is feature-level fusion, which is the most fundamental and widely applied fusion strategy. Its core principle involves separately extracting EEG spectral features (conventional bandwidth parameters, 1/f aperiodicity indices, microstate indicators) and dynamic fMRI network features (dynamic connectivity strength, network stability, modular efficiency), and then constructing multidimensional joint feature biomarkers through normalisation, feature selection and weighted fusion. This strategy is technically mature, computationally efficient and highly interpretable; it effectively compensates for the dimensional limitations of single-modality indicators, enhances the sensitivity and specificity of early injury screening, and is well-suited to routine clinical testing scenarios (82).

Secondly, source imaging fusion—a spatially precise fusion strategy—relies on EEG source imaging technology to invert scalp EEG signals to cortical source regions, thereby obtaining the spatial distribution characteristics of cortical neuronal electrical activity. This is then spatially registered and fused with functional activation and network connectivity data from dynamic fMRI (83). This strategy addresses the core challenge of insufficient spatial resolution in EEG, enabling a dual assessment of “precise localisation of cortical electrical activity and quantification of brain network function”. It can accurately identify the brain regions responsible for early brain injury and abnormal network pathways, thereby supporting injury localisation, mechanism analysis and the selection of targeted rehabilitation targets. It is frequently used for the detailed assessment of complex and subtle brain injuries.

Thirdly, deep learning fusion represents a cutting-edge intelligent fusion strategy. Relying on AI models such as convolutional neural networks, Transformers and multimodal fusion neural networks, it automatically mines deep-level associative features between EEG time-series electrophysiological data and fMRI spatial network data. Without the need for manual feature selection, it enables end-to-end intelligent diagnosis and injury grading. This strategy is capable of capturing faint abnormal coupling signals that cannot be identified through manual analysis, significantly improving the detection rate of early-stage micro-injuries, whilst also enabling automatic stratification of injury severity and intelligent prediction of prognostic risk. However, this strategy suffers from issues such as poor model interpretability, reliance on large datasets, and susceptibility to overfitting; it is currently in a transitional phase from research to clinical application (84).

A comprehensive comparison of the three strategies in terms of technical maturity, clinical accessibility and screening accuracy indicates that feature-level fusion is currently the most pragmatic option for many clinical settings—it has a low technical threshold, high computational efficiency and strong interpretability, and can be rapidly deployed within existing clinical workflows (85). Source imaging fusion can serve as a supplementary tool for precise localisation in complex cases where feature-level fusion is inconclusive (86). Deep learning fusion represents the future direction; however, it can only be introduced into clinical practice once issues of overfitting and generalisation have been resolved using large, multi-centre databases (*n* > 1,000) (87). We do not claim that feature-level fusion is universally optimal. Rather, it may be preferable in settings where interpretability, computational efficiency, and ease of deployment are prioritised, whereas other strategies may be better suited to specific clinical questions or data modalities. Deep learning fusion, in particular, carries additional risks, including dataset shift, limited interpretability, potential algorithmic bias, privacy concerns, and unclear regulatory pathways. These issues must be addressed before clinical deployment. In particular, prospective validation, external testing in independent cohorts, and regulatory approval pathways for AI-based biomarkers remain underdeveloped.

### 4.3 Diagnostic advantages of multimodal combined biomarkers in early-stage damage

Multimodal combined biomarkers, constructed on the basis of spectral parameters and dynamic network indicators, integrate the high temporal resolution and micro-level electrophysiological quantification advantages of EEG with the high spatial resolution and macro-level network visualisation advantages of dynamic fMRI, offering significant diagnostic advantages over single-modality biomarkers. Preliminary evidence suggests that multimodal markers may improve classification performance in selected research settings (85). However, most studies are small, heterogeneous, and proof-of-concept. It remains unclear whether these models provide incremental predictive value over established clinical variables or improve clinical decision-making and outcomes (88). Secondly, the assessment is more comprehensive, potentially enabling a three-dimensional evaluation of damage spanning “microscopic electrophysiology—mesoscopic brain region activity—macroscopic whole-brain networks”. This not only determines the presence of damage but also quantifies its severity, localises the pathways of damage, and assesses the overall level of impaired brain function.

Thirdly, it addresses the need for assessing the reversibility of early-stage damage. Combined multimodal indicators may capture abnormalities that are not detectable by conventional structural imaging and may help track recovery trajectories. However, current evidence does not establish that these markers can reliably distinguish reversible from irreversible damage. Such claims require longitudinal, histologically or clinically validated evidence, and may inform future research on intervention timing rather than current clinical decisions. Fourthly, it offers greater value for prognostic assessment. Whilst a single indicator can only reflect damage along a single dimension, multimodal combined markers show a stronger correlation with patients' long-term cognitive recovery, neurological rehabilitation and risk of recurrence. These associations suggest potential for risk stratification and prognostic enrichment (89), but prospective validation is required before clinical use. Existing research confirms that multimodal fusion strategies can significantly enhance the efficacy of diagnosis and prognostic assessment. For example, Shi et al. (84). used a deep learning fusion model based on synchronised EEG-EMG and fMRI to predict motor recovery following stroke, achieving an accuracy of 80.31% and an AUC of 0.8665, which was significantly superior to single-modality fMRI (71.69%) and single-modality EEG-EMG (68.00%) models. Vedaei et al. also found that the classification accuracy of multimodal models for chronic mTBI could be improved from 79 to 91.67 per cent for monomodal approaches to 95.83 per cent (90). The above evidence indicates that multimodal fusion is one of the technical approaches with the greatest clinical potential in the current field of early-stage brain injury screening.

## 5 Application in typical early-stage brain injury

While the potential of multimodal fusion is promising, its clinical value must be evaluated within specific subtypes. Given the differences among these subtypes, biomarker specificity and generalisability across subtypes should not be assumed, and each subtype may require separate validation. The following sections therefore discuss each subtype separately, with attention to subtype-specific evidence.

To review the potential translation value of dynamic fMRI-EEG multimodal fusion in representative early-stage brain injury subtypes, we conducted a literature search in PubMed and Web of Science using keywords including “mTBI”, “concussion”, “silent stroke”, “subclinical brain injury”, “EEG spectral analysis”, “dynamic fMRI”, and “multimodal fusion”, from database inception to February 2026 (final search: February 2026). This is a narrative review rather than a systematic review. This literature search was performed for narrative synthesis purposes only and does not follow systematic review methodology (e.g., PRISMA guidelines); no formal screening, data extraction, or quality appraisal was conducted. Studies were considered if they reported quantitative EEG or dynamic fMRI metrics in early-stage brain injury populations, and excluded if they focused solely on structural imaging or severe TBI. Based on this search strategy, we summarize the representative findings and clinical applications in the following three typical scenarios. Before interpreting these findings, however, it is important to note that an association between a biomarker and disease severity or outcome does not imply that it can be used for screening, treatment selection, or prognosis in clinical practice. These are distinct levels of evidence. Accordingly, in the following sections, we distinguish between statistical associations, classification performance, incremental predictive value, and clinical utility.

### 5.1 Mild traumatic brain injury (concussion) and return-to-sport screening

Building on the screening advantages of the aforementioned multimodal fusion technology, we have integrated the complete pathway for mTBI, from initial screening to clinical decision-making, to form an objective and quantifiable assessment process, as shown in Figure 4. This pathway begins with high-risk populations, starting with rapid screening via bedside EEG to detect subtle spectral abnormalities; and for individuals who test positive, dynamic fMRI is then used for precise localisation to assess dynamic connectivity changes in core brain networks; finally, multi-modal fusion assessment may enables stratified diagnosis of the injury and decision-making regarding return to sport, aiming to provide clinicians with a structured and objective assessment tool.

**Figure 4:**
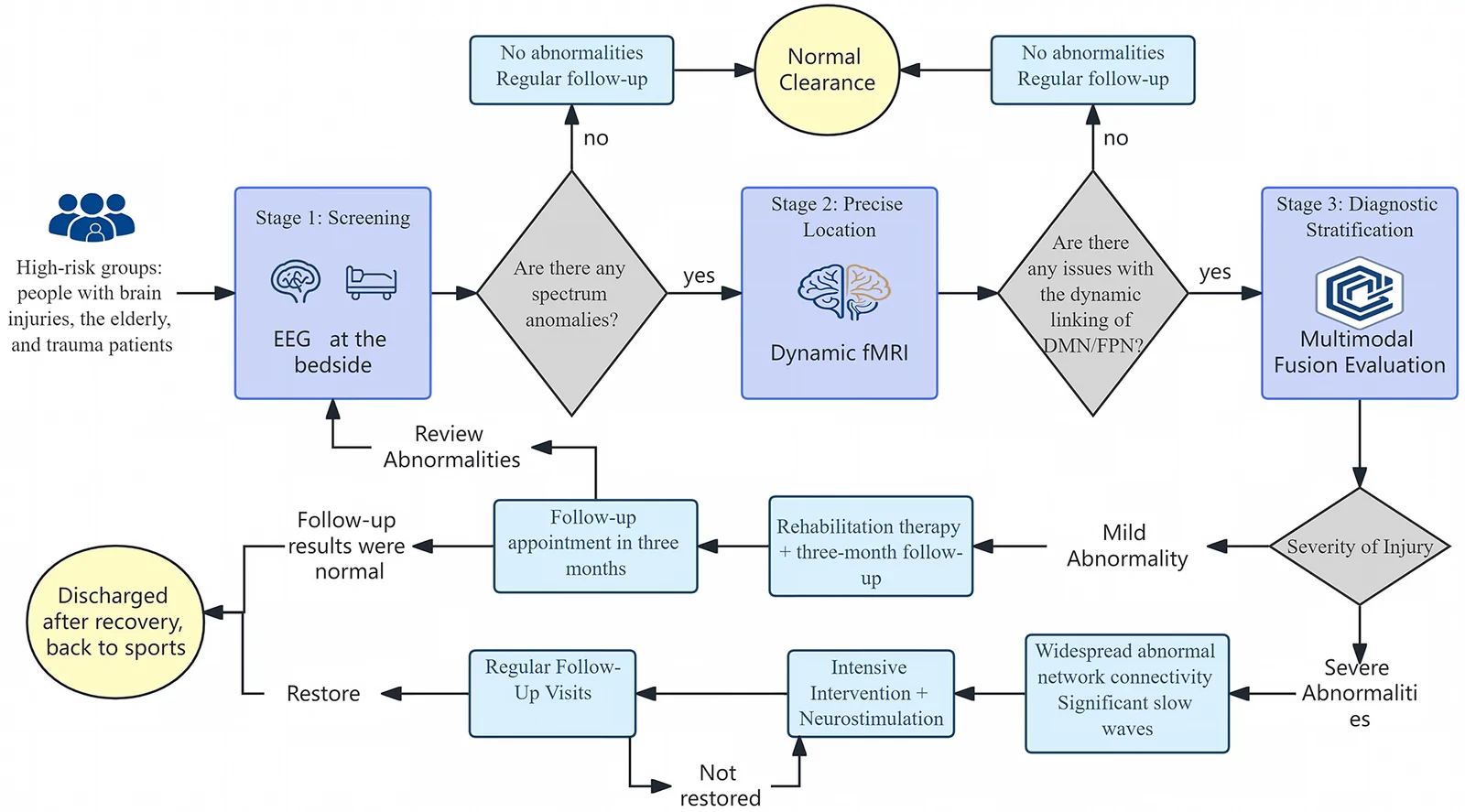
Screening and clinical decision-making pathway for mild traumatic brain injury based on multimodal fusion.

Mild traumatic brain injury (mTBI, concussion) is the most common type of early-stage brain injury, often caused by sports-related impacts or minor trauma. The vast majority of patients show no significant abnormalities on cranial CT or structural MRI scans, yet they exhibit subtle EEG spectral slowing and dynamic disruptions in brain networks (91). Returning to sport before full recovery is highly likely to lead to serious adverse outcomes, such as secondary brain injury and chronic traumatic encephalopathy. Currently, the field of sports medicine lacks unified and objective assessment criteria for return to sport. Traditional subjective symptom scoring and simple cognitive tests are highly subjective and lack precision (92); multimodal fusion technology provides an objective, quantifiable basis for mTBI screening and return-to-sport assessment.

During acute-phase screening, mTBI patients exhibit significant EEG spectral abnormalities, characterised by an elevated θ/α ratio (19), increased slow-wave activity, a flattened 1/f aperiodic slope, and disrupted microstate transitions (93). Concurrently, dynamic fMRI reveals a significant decline in the dynamic connectivity stability of the DMN and the frontoparietal network (94, 95). Combined multimodal biomarkers may help identify subclinical post-traumatic brain dysfunction and distinguish concussion from simple soft-tissue injury. During follow-up in the recovery phase and return-to-sport assessments, multimodal techniques can dynamically monitor the recovery process of EEG spectral parameters and brain network stability (96). When spectral parameters return to normal levels and dynamic brain network connectivity stabilises, this may suggest restoration of brain function. However, return-to-sport decisions should not rely solely on these markers (97); they may serve as supportive evidence alongside clinical assessment. If indicators remain abnormal, this suggests residual functional impairment, necessitating an extended rehabilitation period to mitigate the risk of secondary injury. This multimodal assessment system is currently being progressively applied to concussion screening and return-to-sport assessments for professional athletes and school-age sports participants.

### 5.2 Early warning of silent ischaemic/subclinical small-vessel brain damage

Silent ischaemic injury and subclinical damage to cerebral small blood vessels are common forms of early cerebrovascular damage in middle-aged and elderly individuals. These conditions present without typical stroke symptoms, and structural imaging struggles to detect minute ischaemic lesions; however, they lead to diffuse cerebral microcirculatory dysfunction and neuronal ischaemia-hypoxia injury. They represent a critical precursor stage to symptomatic stroke and vascular cognitive impairment, and early warning and intervention can significantly reduce the long-term risk of cerebrovascular events (98). The core pathological changes associated with this type of early damage are neuronal E/I imbalance, disrupted electrical activity and decline in brain network function, all triggered by abnormalities in cerebral microcirculation; these findings are highly consistent with characteristics of EEG spectral slowing and dynamic brain network disruption.

Clinical studies indicate that high-risk individuals for subclinical damage to cerebral small blood vessels exhibit significant abnormalities in brain electrical activity—such as increased delta-band power in the prefrontal cortex—prior to the onset of clinical symptoms or structural imaging abnormalities (99). Concurrently, dynamic fMRI can detect reduced whole-brain network efficiency and disrupted network connectivity in deep brain regions (98). Multimodal fusion technology can quantify neuronal ischaemic functional damage via spectral parameters and assess whole-brain functional abnormalities caused by cerebral microcirculatory damage through dynamic network indicators, thereby showing potential for ultra-early warning of occult cerebrovascular damage. Furthermore, the severity of abnormalities in these multimodal indicators correlates positively with the burden of small-vessel brain damage and the risk of cognitive decline. These indicators may inform risk stratification (99), but their screening value remains to be established in prospective studies. They may guide preventive interventions such as early blood pressure reduction, improvement of circulation, and neuroprotection, thereby potentially reducing the risk of stroke.

### 5.3 Risk stratification and rehabilitation prognosis assessment for subclinical brain injury

Subclinical brain injury broadly refers to various types of hidden brain injury characterised by the absence of clinical symptoms, negative structural imaging findings, and the presence of cerebral functional abnormalities alone. It encompasses cerebral functional impairment resulting from mild trauma, chronic cerebral ischaemia, long-term sleep disorders, and chronic fatigue syndrome (100). Individuals with such injuries face a significantly higher risk of long-term cognitive decline, mood disorders, and neurological deficits compared to the healthy population; however, there is a lack of effective risk stratification tools (101). Multimodal fusion technology, with its advantages of comprehensive and quantitative assessment, may help stratify risk in subclinical brain injury, although prospective validation is required (102). Based on the degree of EEG spectral abnormalities and the extent and severity of dynamic brain network disruption, subclinical injury is classified into mild, moderate and severe risk levels, providing a basis for personalised intervention.

In terms of rehabilitation prognosis assessment, multimodal indicators may help predict rehabilitation outcomes and long-term prognosis in patients with subclinical brain injury (96). Patients with mild functional abnormalities can rapidly return to normal EEG spectral parameters and dynamic brain network indicators following standardised rehabilitation intervention, with a favourable prognosis; whereas patients with severe subclinical brain injury (103), characterised by significantly abnormal 1/f aperiodic parameters and widespread disruption of the whole-brain network, face significant challenges in spontaneous recovery and are at high risk of long-term cognitive decline and symptom manifestation, necessitating enhanced (104), individualised rehabilitation intervention. Furthermore, multimodal technology enables dynamic monitoring of brain functional recovery during rehabilitation, allowing for real-time assessment of treatment efficacy (102). This provides objective, quantifiable evidence for the dynamic adjustment and optimisation of rehabilitation programmes, thereby significantly enhancing the accuracy of neurorehabilitation.

Biomarker specificity and generalisability across subtypes. Although a shared analytical framework may be applied across mTBI, silent stroke, and subclinical brain injury, the underlying pathophysiology differs substantially (1). Biomarkers developed in one subtype may not generalise to others. For example, spectral slowing and network disorganisation are not specific to early brain injury and are observed in other neurological and psychiatric conditions (85). Future studies should report specificity alongside sensitivity and validate candidate biomarkers in independent, subtype-specific cohorts (88).

## 6 Clinical translation and current limitations

Having discussed the specificity and generalisability of biomarkers across subtypes, we now turn to the clinical translation of multimodal fusion in neurorehabilitation.

### 6.1 Applications in neurological rehabilitation: injury stratification, prognosis prediction, and guidance for neurostimulation

#### 6.1.1 Established evidence

At present, there is no established evidence that dynamic fMRI-EEG fusion improves rehabilitation outcomes. Existing evidence is limited to associations between multimodal markers and injury severity or recovery potential. For example, multimodal markers have been associated with clinical recovery trajectories after sports-related concussion, but these findings do not establish clinical utility (96).

#### 6.1.2 Emerging evidence

Emerging evidence suggests that multimodal markers may help stratify injury severity, estimate rehabilitation potential (105), and identify high-risk groups with poor prognosis (102). However, most studies are small, heterogeneous, and lack external validation. Prospective validation is required before these markers can be used in rehabilitation planning. Multimodal approaches have also been used to predict recovery after stroke. Antonioni et al. combined EEG measures with kinematic data and found that both were correlated with upper limb motor recovery at 4 weeks post-stroke (106). Pierella et al. integrated kinematic, muscular, and brain activity using a multivariate approach and identified physiological biomarkers with potential for patient stratification and rehabilitation protocol design (107). Although stroke is a distinct disease, it shares several key features with early brain injury, including substantial individual differences, a dissociation between lesion burden and functional impairment, and the need to predict recovery over time. These findings support the rationale for multimodal fusion in early brain injury, while also highlighting the need for external validation, standardised outcome definitions, and prospective multi-centre designs.

#### 6.1.3 Future hypotheses

A future hypothesis is that multimodal fusion could guide precision neurostimulation by combining EEG-derived electrophysiological targets with fMRI-derived network targets (83). This could theoretically enable personalised neuromodulation and a closed-loop rehabilitation system comprising assessment, intervention, follow-up, and optimisation (102). However, no clinical trial has yet demonstrated that such a closed-loop system improves outcomes. This remains a future direction rather than an established clinical application.

### 6.2 Key bottlenecks in clinical translation and methodological reflections

The primary bottleneck lies in the challenges of standardisation and data silos. Currently, there is a lack of consensus amongst different research teams regarding EEG spectral pre-processing (e.g., denoising algorithms, reference electrode selection), 1/f parameter calculation models (e.g., parameter settings for the FOOOF algorithm), dynamic fMRI network construction (e.g., sliding window width, number of states K), and multimodal fusion strategies (e.g., feature-level vs. model-driven approaches) (108). This results in a lack of universality and comparability in the quantification of indicators; there are no industry-standardised diagnostic thresholds, making it difficult to establish a standardised clinical screening system. These methodological differences can substantially affect between-study comparability and may compromise the reproducibility of findings. If such parameters are ultimately proposed as quantitative clinical biomarkers, external validation and harmonised acquisition/analysis pipelines will be essential to ensure their reliability and generalisability across populations and settings. At the same time, existing normal reference thresholds are predominantly based on Western populations, whilst there are known racial differences in the background EEG rhythms and brain network developmental trajectories of the Chinese population; direct application of these thresholds will lead to a decline in screening accuracy. Furthermore, the absence of a large-sample, multi-centre database of multimodal parameters for early brain injury in the Chinese population severely hampers the localised clinical translation of this technology (109). In addition, existing studies are predominantly single-centre, small-sample designs, with samples concentrated on middle-aged and young patients; there is a lack of stratified research involving the elderly, children and those with comorbidities, resulting in insufficient model generalisation ability (85).

The second major obstacle relates to clinical accessibility. Simultaneous data acquisition equipment is costly and requires a specialised MRI-compatible EEG system; whilst asynchronous acquisition is less expensive, the complete collection of multimodal data still necessitates multiple patient visits, entailing significant time and financial costs. Currently, the entire process—including multimodal data acquisition, pre-processing, feature fusion and model analysis—requires operation by specialised technical personnel. The lengthy testing and analysis cycles are severely at odds with the needs for rapid screening in A&E departments and high-volume health screening in primary care settings, thereby limiting the technology's large-scale implementation.

A deeper-seated challenge lies in methodological issues and a crisis of interpretability. Dynamic fMRI and EEG time-series metrics are highly dimensional and involve large volumes of data; deep learning fusion models are highly prone to overfitting during training on small samples, resulting in artificially high accuracy on the training set but insufficient generalisation ability in real-world scenarios. Furthermore, confounding factors such as age, gender, sleep quality, emotional state, underlying medical conditions, pharmacological interventions and the testing environment all significantly influence EEG and network metrics; existing studies struggle to fully standardise baseline conditions, resulting in poor reproducibility of results. More fundamentally, features such as spectral slowing and network disorganisation are also observed in other brain functional disorders and certain psychiatric and psychological conditions; the current lack of specific diagnostic markers makes it difficult to accurately distinguish early-stage brain injury from other brain functional disorders (85).

This lack of specificity has direct implications for screening. Spectral slowing and network disorganisation are not unique to early brain injury and can be observed in epilepsy (110), Alzheimer's disease (111), schizophrenia (112), depression (111), sleep deprivation (113), and medication effects (114). Brain network disorganisation, in particular, has been documented across schizophrenia, epilepsy, stroke, and Alzheimer's disease as a common feature rather than a disease-specific marker (115). Without active control groups and differential diagnosis cohorts, apparent group differences may reflect non-specific markers of brain dysfunction rather than early brain injury. Therefore, future studies should report specificity, positive predictive value, and likelihood ratios alongside sensitivity and AUC (116). For a screening test intended for asymptomatic or mildly symptomatic populations, high specificity is essential to avoid false positives, overdiagnosis, and unnecessary downstream testing.

The comparability of physiological states across asynchronous EEG and fMRI sessions remains an open question, and future work should address state-dependence and temporal instability.

## 7 Summary and future prospects

### 7.1 Summary

Clinical screening for early-stage brain injury has long been hampered by the insidious nature of structural imaging—where results may be “negative” whilst functional impairment is already present. The core pathological mechanism lies in the disruption of microscopic neuronal electrical activity caused by an imbalance between cortical excitation and inhibition, and the resulting dynamic functional reorganisation of the whole-brain neural network. EEG spectral parameters (including the 1/f aperiodicity index), with their millisecond-level temporal resolution, can precisely quantify abnormalities in neuronal discharge rhythms and E/I shifts, offering unique advantages for dynamic bedside screening; dynamic fMRI, with its millimetre-level spatial resolution, effectively characterises the dynamic connectivity destabilisation and reconstruction patterns of the DMN, SN and FPN. The two approaches are naturally complementary in the spatio-temporal dimensions, whilst the dynamic-spectral coupling (DSC) framework proposed in this paper further elucidates, at a mechanistic level, the hypothesised cascade from 'spectral abnormalities' to “network drift”, providing a potential theoretical anchor for multimodal fusion techniques. Building on this foundation, three strategies—feature-level fusion, source imaging fusion and deep learning fusion—have established a three-dimensional screening system linking “microscopic electrophysiology” with “macroscopic network function” across three depth levels: “clinical accessibility”, “localisation of complex cases” and “end-to-end intelligent diagnosis”. Current evidence suggests that this multimodal fusion approach may offer potential advantages over single modalities in some settings, although existing studies are limited by small samples, methodological heterogeneity, and lack of external validation. It may represent a promising direction, but further prospective, multi-centre validation is required before it can be considered a robust clinical tool.

### 7.2 Future directions: from proof of concept to clinical implementation

To overcome the aforementioned three-fold barriers, a coordinated approach is required across four dimensions: laying the foundations through standardisation, scaling down tools for wider access, enhancing efficiency through intelligence, and establishing a closed-loop clinical system.

Firstly, establish unified data standards and localised reference norms. There is an urgent need to conduct multi-centre collaborative studies to standardise data pre-processing, feature extraction and model evaluation criteria, and to establish a multi-modal parameter database specific to the Chinese population, encompassing healthy controls and patients with various types of early-stage brain injury, thereby laying the foundation for industry-wide diagnostic thresholds and technical specifications. Secondly, promote the development of lightweight tools and their deployment in grassroots settings. Research and develop portable, high-precision EEG devices and lightweight dynamic network analysis algorithms to lower the barriers to equipment and operation, enabling screening technologies to be extended to primary healthcare facilities, communities and home monitoring settings. Third, future studies should build a comprehensive AI-assisted diagnosis and treatment system. Drawing on a multi-centre, large-sample database, we will develop highly interpretable AI-integrated diagnostic systems to achieve end-to-end automation—from data pre-processing and injury grading to prognosis prediction—thereby reducing reliance on specialist technical personnel. Fourthly, future studies should deepen research into underlying mechanisms and establish a closed-loop rehabilitation system. We will thoroughly investigate the mechanisms linking multimodal biomarkers to the efficacy of neurorehabilitation and long-term prognosis, and construct a personalised rehabilitation closed-loop system comprising “screening—stratification—intervention—follow-up”. This will drive the full-chain clinical translation of early brain injury management from simple functional screening to precision prevention and personalised rehabilitation interventions, ultimately achieving the overarching goal of improving patients' long-term prognosis.
